# How the Cervical Microbiota Contributes to Cervical Cancer Risk in Sub-Saharan Africa

**DOI:** 10.3389/fcimb.2020.00023

**Published:** 2020-02-12

**Authors:** Cameron Klein, Crispin Kahesa, Julius Mwaiselage, John T. West, Charles Wood, Peter C. Angeletti

**Affiliations:** ^1^Nebraska Center for Virology, School of Biological Sciences, University of Nebraska-Lincoln, Lincoln, NE, United States; ^2^Ocean Cancer Institute, Dar es Salaam, Tanzania

**Keywords:** HPV, HIV, cervical cancer, microbiome, sub-saharan Africa

## Abstract

Despite ongoing efforts, sub-Saharan Africa faces a higher cervical cancer burden than anywhere else in the world. Besides HPV infection, definitive factors of cervical cancer are still unclear. Particular states of the cervicovaginal microbiota and viral infections are associated with increased cervical cancer risk. Notably, HIV infection, which is prevalent in sub-Saharan Africa, greatly increases risk of cervicovaginal dysbiosis and cervical cancer. To better understand and address cervical cancer in sub-Saharan Africa, a better knowledge of the regional cervicovaginal microbiome is required This review establishes current knowledge of HPV, HIV, cervicovaginal infections, and the cervicovaginal microbiota in sub-Saharan Africa. Because population statistics are not available for the region, estimates are derived from smaller cohort studies. Microbiota associated with cervical inflammation have been found to be especially prevalent in sub-Saharan Africa, and to associate with increased cervical cancer risk. In addition to high prevalence and diversity of HIV and HPV, intracellular bacterial infections such as Chlamydia, Gonorrhea, and *Mycoplasma hominis* are much more common than in regions with a low burden of cervical cancer. This suggests the prevalence of cervical cancer in sub-Saharan Africa may be partially attributed to increased cervical inflammation resulting from higher likelihood of cervical infection and/or microbial dysbiosis.

## Introduction

In sub-Saharan Africa, cervical cancer risk is far greater than in developed countries. Human Papillomavirus (HPV) is a major health concern worldwide, contributing to an estimated 4.8% of all cancers (Forman et al., 2012). This percentage drastically increases in less developed regions of the world, with HPV attributing to 14.2% of all cancers in sub-Saharan Africa (Forman et al., 2012). It is well-established that HPV is the causative agent of cervical cancer (De Vuyst et al., 2013). In 2013, 485,000 women were diagnosed with cervical cancer, and 236,000 deaths were attributed to cervical cancer. It is estimated 1 in 70 women worldwide will develop cervical cancer before reaching 79 years of age (De Vuyst et al., 2013; Fitzmaurice et al., 2015). Cervical cancer disproportionately affects sub-Saharan Africa, where 9% of the world's female population accounts for 14% of the world's incident cervical cancer and 18% of cervical cancer related deaths (De Vuyst et al., 2013). This results in a cervical cancer mortality risk of 2.7%, about 70% higher than the second highest region: South-Central Asia (De Vuyst et al., 2013). In 2013, cervical cancer was the most common cause of cancer death in women in 46 of 54 sub-Saharan African countries (85%) (Fitzmaurice et al., 2015). Only 5 countries outside of sub-Saharan Africa count cervical cancer as the most common cause of cancer death in women. Despite current efforts against cervical cancer in sub-Saharan Africa, it is estimated the number of cervical cancer cases will continue to rise, highlighting the need to bring sub-Saharan Africa up to modern standards for HPV treatment and prevention, and to understand the factors contributing to cervical cancer in the region (Williamson, 2015).

Sub-Saharan Africa faces many unique issues regarding cervical cancer. Screening and prevention practices, sociocultural aspects, HPV genotype prevalence, HIV prevalence, HIV treatment, sexually transmitted infections (STIs), and the composition of the cervicovaginal microbiome are all important factors, which differ in sub-Saharan Africa compared to more developed regions. While some of these factors have been correlated with increased cervical cancer incidence, it is unclear how or if they contribute to HPV pathogenesis.

Screening is the key to early detection and treatment of cervical cancer and identifying at-risk populations. Cervical cancer rates in developed countries with screening and treatment programs, have cervical cancer rates below 10 per 100,000 women. In The USA, coverage of these screening programs within a 48 month period is 94% of women ages 25 to 29 years, decreasing at older ages to 69% at 45 to 49 years and 55% at 60 to 64 years (Cuzick et al., 2014). In countries without screening programs, cervical cancer rates are significantly higher (Fitzmaurice et al., 2015). In sub-Saharan Africa, screening methods and their efficiency vary significantly. Because of this, coverage is difficult to estimate and largely based on speculation. The most common cervical cancer screening method in sub-Saharan Africa is visual inspection with acetic acid (VIA) rather than the pap smear, the preferred method in developed countries. VIA is cost effective, but is known to be less specific since it depends on visual recognition of lesions, whereas the pap smear identifies abnormalities at the cellular level (1999). The major factor contributing to the high incidence of cervical cancer in sub-Saharan Africa is the lack of reliable cervical cytology screening. Historically, introduction of population screening programs has reduced cervical cancer incidence by 25–77% (Gustafsson et al., 1997). Establishing better screening programs is a necessary step toward reducing the burden of cervical cancer in sub-Saharan Africa, however this alone is not enough to address the issue. Ignoring the contribution of current screening efforts in sub-Saharan Africa, even the most drastic decrease in cervical cancer after implementation of population screening seen historically (77%) would not bring cervical cancer rates as low as those in developed countries with population screening. This emphasizes the importance of understanding and addressing what other factors in sub-Saharan Africa are contributing to cervical cancer.

Besides screening, most factors correlated with developing cervical cancer relate to the cervical immune microenvironment. Recent research into the cervicovaginal microbiome has uncovered intricate relationships between the bacterial microbiota, HPV, HIV, and cervical cancer (Godoy-Vitorino et al., 2018; Huang et al., 2018; Klein et al., 2019). These relationships suggest that certain cervicovaginal microbes, or the microenvironment created by certain microbes, are cofactors of cervical cancer progression. HIV is a well-studied factor in sub-Saharan Africa, which influences the cervical microbiota. Cervical cancer is classified as an AIDS-defining cancer due to greatly increased risk among HIV positive individuals with low T cell count. Despite extensive study of the prevalence and impact of HIV in sub-Saharan Africa, the exact mechanism by which HIV infection contributes to HPV driven cervical cancer remains unclear. A better understanding of other correlated factors will help clarify the mechanisms which drive cervical cancer, and address how to bring sub-Saharan Africa in line with other regions. STI screening of genital tract infections like chlamydia and gonorrhea has found they are much more prevalent in sub-Saharan Africa, while, metagenomic studies of the cervicovaginal microbiome have shown significant differences between the commensal and non-commensal components of sub-Saharan African microbiomes when compared with low cervical cancer risk areas. Considering such infections have been associated with pre-cancerous lesions, it is likely these differences, in part, account for sub-Saharan Africa's increased cervical cancer risk (Onywera et al., 2019).

Defining differences in cervical microbiota by geographic location, HIV status, and cervical cytology using compiled published data is difficult due to major differences in cohort makeup, cohort size, sampling and sequencing techniques, and other issues. The cervical microbiota varies greatly between individuals. Factors such as age, race, menstrual phase, and lifestyle have all been shown to affect the microbiome. Controlling for such a large number of factors is difficult, which has hindered the discovery of definitive microbiota. Furthermore, the microbiota of the cervix has been shown to be significantly different than that of the vagina, thus studies which sample the cervicovaginal microbiome do not best represent the microenvironment at the site of cervical transformation (Koedooder et al., 2019). For these reasons, the results of studies are considered individually in this review, so that each speaks only for the niche represented by its cohort. To better understand and address the relationship between HPV, HIV, cervical microbiota, and increased cervical cancer risk, a better understanding of the unique sub-Saharan African environment is needed. Here, we discuss current knowledge in each of these areas, highlighting factors especially prevalent in sub-Saharan Africa which may drive HPV-dependent cervical cancer.

## HPV Genotypes

The HPV family includes more than 200 genotypes, over 45 of which are known to infect the anogenital region. The regional prevalence and oncogenic potential of HPV genotypes varies significantly. Fifteen anogenital HPVs are classified as high-risk for development of cervical cancer (Guan et al., 2012). Among these, HPV16 and 18 are the predominant oncogenic genotypes, causing approximately 70% of cervical cancer cases globally (Ogembo et al., 2015). The relative oncogenic potential of HPV 16 and 18 has been shown to be markedly higher than that of other genotypes, followed by 45, 69, 58, 31, 33, 34, 67, 39, 59, 73, and 52 by decreasing oncogenic potential (Bernard et al., 2013). Of the global HPV burden, 22.5% of HPV infections are estimated to be produced by HPV-16, however, a significant inverse correlation has been observed between overall HPV prevalence and the contribution of HPV-16, with the lowest HPV16 proportions in the regions with the highest HPV prevalence (Bruni et al., 2010). Not surprisingly, sub-Saharan Africa has been shown to have the lowest HPV-16 contribution to total HPV infections in women with normal cervical cytology when compared to other regions, with estimates of 13.7, 11.3, and 11.1% for Southern, Eastern, and Western Africa, respectively (Bruni et al., 2010). This correlation is even more pronounced in cervical cancer, where HPV16 and 18 are less frequent in sub-Saharan Africa than in the rest of the world (49.4 vs. 62.6%), while HPV18 and HPV 45 are two times more frequent (19.3 vs. 9.4% and 10.3 vs. 5.6%) (Ndiaye et al., 2012). After HPV16 and 18, the most prevalent genital HPV genotypes vary between sub-Saharan Africa countries. Overall, HPV 52, 35, 58, 33, 31, 45, 53, and 51 are the most prominent non-16/18 genotypes in sub-Saharan Africa (Forman et al., 2012; Abate et al., 2013; Olesen et al., 2013; Adler and Wallace, 2014; Boumba et al., 2014, 2015; McDonald et al., 2014; Mihret et al., 2014; Bateman et al., 2015; Lebelo et al., 2015; Okonko and Ofoedu, 2015; Padalko et al., 2015; Pirek et al., 2015; van Aardt et al., 2015). When comparing prevalence with high-income regions, HPV 52, 58, 33, and 45 stand out as especially prevalent in sub-Saharan Africa (Bruni et al., 2017). Because these HPV genotypes are only common in Sub-Saharan Africa, they have not been as well-researched as globally prevalent HPVs such as 16 and 18. Potential differences in pathogenesis in such genotypes may contribute to increased cervical cancer in Sub-Saharan Africa, where a larger percentage of cervical cancer cases are attributed to non-HPV16/18 genotypes.

Accurate detection and identification of HPV genotypes depends upon the genotyping method used. Most large studies use one of several established genotyping assays, however more recent studies using sequencing-based identification of HPV genotypes have found that genotyping assays may only detect as little as 49% of those able to be detected with sequencing (Ndiaye et al., 2012). The bias introduced by genotyping assays may downplay the significance of certain HPV genotypes in Sub-Saharan Africa, especially those which have not been well-researched, such as HPV 34, 67, 69, and 73. Additionally, the extensive sequence variation within HPV genotypes, which has been demonstrated to be especially severe in Sub-Saharan Africa, is not accounted for by genotyping assays as it is in sequenced-based approaches, and may be of clinical importance. Sequence variation of HPV may also contribute to reduced efficacy of HPV vaccination in Sub-Saharan Africa, while increased genotypic diversity of HPV almost certainly does. A more complete understanding of the microbiome/HPV interactions is necessary before an effective microbiome-based intervention can be developed.

Concurrent cervical infection with multiple HPV genotypes is common in Sub-Saharan Africa, however it is not clear if this represents a specific mechanism driving pathogenesis. Data from developed regions suggests multiple infection with HPV decreases in cervical cancer cases, however studies in Sub-Saharan Africa suggest coinfection is more prevalent in cervical cancer and may exacerbate HPV pathogenesis. A study of South African women with cervical cancer found that 65% were coinfected with at least two HPV genotypes (Lebelo et al., 2015). Of the coinfected cervical cancer cases, 90.4% included HPV16, suggesting infection with other HPV genotypes may contribute to HPV16 driven cervical cancer. Similar results were found in a study of women in the Democratic Republic of Congo (Boumba et al., 2014). Further work has shown higher HPV16 viral loads in 70.3% of HPV16 coinfected samples (Lebelo et al., 2015). In a separate study, Cameroonian women with normal cervical cytology and multiple HPV infections were found to be about 10% more likely to develop cervical lesions within a year when compared to women infected with a single HPV type (Pirek et al., 2015). These results suggest a synergistic effect driving HPV replication and cervical cancer pathogenesis in cases of multiple HPV infection.

In contrast, other publications have suggested HPV16 may be more sensitive to attack from other genotypes, and thus may be at higher risk of competition when there is more immune suppression (Menon et al., 2016). A meta-study examining global HPV prevalence found that multiple HPV infections were, on average, 6% more common in women with normal cytology than in those with cervical cancer (Bernard et al., 2013). Sub-Saharan Africa was the least represented region by studies included in this analysis, allowing for the possibility that synergistic effects in cases of multiple infection are primarily found in the genotypes most prevalent in Sub-Saharan Africa. Further research focusing on the long-term oncogenic potential of different combinations of HPV genotypes, is necessary.

## HIV

**Figure 1:**
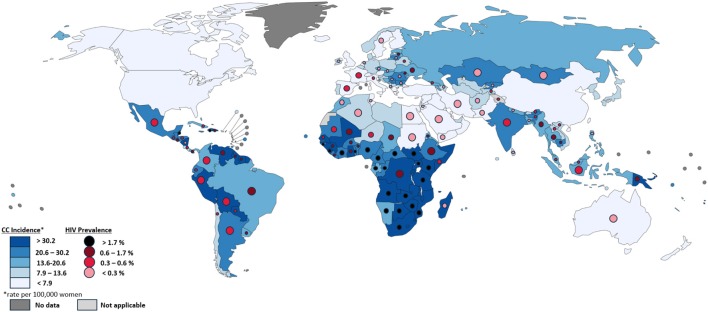
Comparison of cervical cancer incidence and HIV Prevalence by country. Each country is colored by cervical cancer incidence per 100,000 women, as described in the bottom left, based on data from GLOBOCAN 2012. Circles within each country's borders are colored by HIV prevalence, as described in the bottom left, based on data from UNAIDS (2016). Differences in the size of circles within countries is only for visibility and does not signify anything meaningful. Countries without HIV prevalence circles did not have such data available. Map produced by IARC.

HIV is the best studied co-factor to cervical cancer and has been strongly linked to severe HPV pathogenesis. The association between severe HIV pathogenesis and cervical cancer has classified cervical cancer as an “AIDS-defining cancer.” In addition to increasing cervical cancer risk, evidence suggests that high HIV prevalence also contributes to increased prevalence and circulation of HPV (Williamson, 2015). Similarly, the widespread prevalence of multiple HPV infections has been shown to contribute to the spread of HIV by increasing susceptibility of HIV acquisition. Multiple studies have shown that immune response to HPV increases HIV-susceptible cells in both male and female genital tracts, increasing the opportunity for an initial infection to occur based on the local immune microenvironment (Averbach et al., 2010; Tobian et al., 2013; Williamson, 2015). The regional relationship between HIV prevalence and cervical cancer is shown in Figure 1, which demonstrates associations between the two globally, highlighting the exceptionally high rates seen in Sub-Saharan Africa.

Risk factors and predictors of cervical cancer are also increased in HIV+ individuals. HPV infection, abnormal Pap smears, and high-grade lesions are significantly more common in HIV+ women (Adler and Wallace, 2014; Salazar et al., 2015). In addition to the increased rate of productive HPV infection, HIV is associated with a higher risk of progression from subclinical to clinical HPV disease (Williamson, 2015). Higher HPV viral loads are associated with increased risk of abnormal cervical cytology, and are seen among those co-infected with HIV, indicating this may in part be due to an undefined mechanism by which HIV infection influences HPV viral replication factors (Depuydt et al., 2012; Wang et al., 2013; Hanisch et al., 2014; Mbulawa et al., 2014). A likely factor in this is a decrease in T-cell surveillance controlling HPV replication with decreasing CD4+ cell count as HIV progresses. Multiple studies have shown an increase in HPV detection, squamous intraepithelial lesions, and cervical intraepithelial neoplasia in individuals with AIDS (less than 200 CD4+ cells per μl serum) (Hanisch et al., 2013; Ezechi et al., 2014; Memiah et al., 2015; Menon et al., 2016). Identifying which aspects of the local and systemic effects of HIV infection contribute to progression from chronic HPV infection to cervical cancer is crucial to understanding the burden of cervical cancer in Sub-Saharan Africa. Current knowledge suggests effects on the cervical immune microenvironment may be key in this process.

In HIV+ populations, there is a shift in prevalence of HPV genotypes, favoring high-risk HPVs (Ezechi et al., 2014). The reasoning for greater prevalence of certain HPV genotypes in HIV+ individuals is not currently well-understood. The influence of HIV may help explain why coinfection of multiple HPV genotypes is associated with cervical cancer in sub-Saharan Africa, but not elsewhere. Studies from several Sub-Saharan African countries have identified a greater number of multiple HPV infections among HIV-positive women (Akarolo-Anthony et al., 2013; Maranga et al., 2013; Adler and Wallace, 2014; McDonald et al., 2014; van Aardt et al., 2015). A study of South African females with cervical cancer found multiple HPV infections in 8% of HIV- women and 27% of HIV+ women (van Aardt et al., 2015). In a study of South African adolescent females, the prevalence of multiple infections was found to be much higher in both HIV positive and negative individuals, with 22% percent prevalence in HIV- and 68.6% in HIV+ (Adler and Wallace, 2014). Only 18.8% of all adolescents in this study had an abnormal pap smear, and none of them were diagnosed with cervical cancer. This supports the idea that exposure to many HPV types occurs early after sexual debut, with certain genotypes becoming dominant by the time HPV pathogenesis reaches cervical cancer. Potentially, infection with “accessory” HPV genotypes contributes to the early pathogenesis of a primary high-risk HPV either directly or through manipulation of the immune microenvironment, leading to increased replication and eventual faster or more frequent development of cervical cancer. These “accessory” HPV infections may then be cleared by a competent immune response, which may explain why multiple infection decreases in HIV- cervical cancer cases, but not in HIV+. Based on current evidence, it is yet unclear whether early multiple HPV infections expedite progression to cervical cancer. A short-term longitudinal study (16 months) was unable to find any additive or synergistic effect of multiple infection on development of cervical lesions, noting that increased frequency of cervical lesions was associated with infection of a single high-risk HPV. Cervical cancer development occurs over a period of decades however; looking at such a narrow time frame means these results may be a consequence of only observing cytological effects in infections in which a high-risk HPV was already established and progressing toward cervical cancer, not early interactions which eventually contribute to lesions (Salazar et al., 2015). Further study focusing on interactions and outcomes in HPV coinfection, especially among young HIV+ women who have not yet developed cervical dysplasia, is desirable to clarify this relationship.

When available, treatment for individuals infected by HIV in sub-Saharan Africa is primarily antiretroviral therapy (ART). Unlike high-income regions, most HIV infected sub-Saharan African individuals go without treatment. ART coverage of HIV infected individuals across sub-Saharan Africa is estimated to be 40.2% or less (UNAIDS, 2016). Studies examining the effects of ART on HPV pathogenesis have had mixed results. While previous studies suggest ART has no significant effect on HPV genotype detection, more recent studies suggest modern ART reduces the prevalence of high-risk HPV's in HIV infected women (Palefsky, 2003; Ezechi et al., 2014; Zeier et al., 2015). This reduction in high-risk HPV prevalence grows with duration of ART use. Besides a reduction of HIV, the effects of ART on cervical microbiota are currently unknown, but may be significant, as several studies have found that ART affects the gut microbiota. ART does not appear to have a significant effect on cervical lesions and tumor development, and only minor effects on limiting progression of lesions and preventing recurrence (Ahdieh-Grant et al., 2004; Paramsothy et al., 2009; Dryden-Peterson et al., 2015; Memiah et al., 2015). A study of Kenyan women found that the spread of ART has been accompanied with a decrease in age-specific cancer risk, however an increase in the number of HPV cancers, which is attributed to an aging HIV+ population rather than to any effect of ART (Memiah et al., 2015). Compared to the risk reduction after ART seen in other AIDS-defining cancers like Kaposi's sarcoma and non-Hodgkin's lymphoma, the risk of cervical cancer is not significantly affected, and recurrence rates remain high with or without treatment (Foulot et al., 2008; Mungo et al., 2013; Russomano et al., 2013; Cobucci et al., 2015). This suggests that HPV depends on immunological status of the host such that ART is only able to indirectly affect HPV pathogenesis, potentially through an effect on circulatory CD4+ cell count and microbiota composition.

## Non-Viral Microbiota

Several studies have proposed that the cervicovaginal microbiota is a co-factor of the development of cervical lesions (Guijon et al., 1992; Kyrgiou et al., 2016; Mitra et al., 2016). The precise mechanism, and the microbes responsible have not been identified, but several common STIs have been associated with cervical cancer individually. Health of the lower female reproductive tract, and its ability to defend against dysbiosis and infection, is directly related to the microbiota present. Its defense mechanisms include antimicrobial peptides, a microbiome dominated by Lactobacilli, and a pH of <4.5. An imbalance in these defenses can result in physiochemical changes, which produce histological alterations of the vaginal mucosa and cervical epithelium (Audirac-Chalifour et al., 2016). Communal differences in the cervical microbiome between sub-Saharan Africa and developed regions have not been well-established, however the prevalence and incidence of pathogenic cervicovaginal microbiota is much higher in sub-Saharan Africa. Among factors associated with preventing or developing cervical cancer, cervicovaginal pathogens are second only to HPV vaccine coverage when comparing differential rates in sub-Saharan Africa and North America (Table 1). Nearly all studies of the cervicovaginal microbiome in sub-Saharan Africa to date have used sequencing of the ribosomal RNA 16s amplicon, which only includes bacteria. Because of this, little is known of the virome or other non-bacterial members of the microbiome outside of targeted screening. Whole genome sequencing (WGS) allows characterization of the microbiome in its entirety and has been shown to be more accurate at the detection of bacterial species and diversity than 16s (Ranjan et al., 2016). RNA sequencing (RNASeq) is another powerful approach to characterize gene expression, which is now being used in microbiome studies. Large scale metagenomic studies of sub-Saharan African populations using WGS is needed to more fully characterize the microbiome and address potential bias introduced by 16s sequencing. These newer methods are likely to improve our understanding of these complex microbial networks.

**Table 1:** Comparison of factors which may influence cervical cancer in sub-Saharan Africa and North America (Koumans et al., 2007; WHO, 2012; Ferlay et al., 2013; CDC, 2014; CDC STD Surveillance, 2015; UN World Fertility Patterns, 2015; WHO Report on the Global Tobacco Epidemic, 2015; Bautista et al., 2016; UNAIDS Global AIDS Update, 2016; UNAIDS Database, 2017; Gonorrhea-CDC Fact Sheet, 2019).

	**North America**	**Sub-Saharan Africa**	**% Difference in sub-Saharan Africa**	**Fold difference**
HPV Vaccine Coverage^a^	35.6%	1.2%	3%	29.7–
*Neisseria gonorrhoeae* Incidence	259.3	5,500	2,121%	21.2+
HIV Prevalence	0.3%	4.38%	1,460%	14.6+
Smoking Prevalence^b^	15.67%	2.3%	15%	6.8–
*Trichomonas vaginalis* Prevalence^c^	3.1%	20.2%	652%	6.5+
cervical cancer Incidence^d^	6.6	35.18	533%	5.3+
*Chlamydia trachomatis* Incidence	456.1	2160	474%	4.7+
Proportion of Adenocarcinoma cervical cancer	18%	5.5%	30.6%	3.3–
Fertility^e^	1.9	4.7	247%	2.8+
HPV Prevalence (NILM)	13.8%	22.9%	166%	1.7+
Anti-Retroviral Therapy Coverage	59%	40.2%	68%	1.5-
Bacterial Vaginosis	29.2%	41.4%	142%	1.4+
HPV 16/18 Prevalence in cervical cancer	71.4%	62.8%	88%	1.1–

a *Women aged 10–20, full-course*.

b *In surveyed females 15+*.

c *Women ages 14+*.

d *Age standardized rate per 100 k women per year*.

e *Children per woman*.

### Non-commensal Microbiota

Chronic inflammation of the cervix is closely associated with developing cervical cancer (Giraud et al., 1998; Skapinyecz et al., 2003; Ilhan et al., 2019). Cervicitis can result from several different conditions, which often are attributed to infection with non-commensal microbes. Pelvic inflammatory disease (PID) in women usually results from bacterial infection of the cervix ascending to the uterus and oviducts, wherein certain bacteria express antigens which induce a chronic inflammatory state. The association observed between PID and cervical cancer is thought to be due to development of a microbiome rich in inflammation-inducing bacteria at the cervix, causing cervicitis. Not surprisingly, PID is more prevalent in HIV-infected women than uninfected (Dehon et al., 2016). The overall prevalence of PID is difficult to define in sub-Saharan Africa, however diagnosis of PID is more than twice as likely to be attributed to a bacterial infection when compared to rates in the developed world (Ross, 2008). This suggests that bacterial infections more often contribute to HPV pathogenesis in Sub-Saharan Africa.

Bacterial vaginosis (BV) is a dysbiosis of cervicovaginal bacteria which, like PID, is associated with cervicitis (Lehtinen et al., 2011; Ogembo et al., 2015). BV alters the cervicovaginal microenvironment, which may increase cervical dysplasia as a result of anaerobic infection producing nitrosamines, which cause cervical inflammation (Lazenby et al., 2014). The microenvironment created by BV has also been identified as a cofactor in the persistence of HPV infection (Gillet et al., 2011; Clarke et al., 2012; Guo et al., 2012; Vriend et al., 2015). Several of the causal bacteria of BV are associated with cervical lesions and/or inflammation. The most common causes of BV are: *Gardnerella vaginalis, Atopobium vaginae, Mobiluncus curtisii, Mobiluncus mulieris, Megasphaera* type 1, M*egasphaera* type 2, *Sneathia sanguinegens, Mycoplasma hominis, Mycoplasma genitalium, Ureaplasma urealyticum, Bacteriodes fragilis*, and bacterial-vaginosis-associated-bacteria (BVAB) 1-3 (Signat et al., 2011; Audirac-Chalifour et al., 2016). Comparative genomic analysis has shown that the shift in microbial diversity as a result of BV is more pronounced in women infected with HIV, suggesting BV is more severe in this population and thus more likely to drive HPV pathogenesis (Spear et al., 2008). In sub-Saharan Africa, BV prevalence is estimated to range from 20 to 50% in reproductive aged women, making it the most common cause of cervicovaginal dysbiosis. This prevalence suggests BV, or the cervical microenvironment created by BV, could be a major contributor to increased malignant HPV pathogenesis in the region, especially among HIV+ women (Msuya et al., 2002; Lewis, 2011; Swanepoel et al., 2013). Further study is necessary to determine if general inflammation caused by conditions like BV and PID is sufficient to promote HPV pathogenesis, or if specific microbes which contribute to the diseases are responsible.

STIs also alter the cervicovaginal microenvironment. Several sexually transmitted microbes have been associated with cervicitis and persistence of HPV infection (Gillet et al., 2011; Lehtinen et al., 2011; Clarke et al., 2012; Guo et al., 2012; Ogembo et al., 2015; Vriend et al., 2015). Among these, *Neisseria gonorrhoeae, Chlamydia trachomatis, Trichomonas vaginalis*, and Syphilis are particularly common in sub-Saharan Africa. Sub-Saharan Africa accounts for a disproportionate 20, 9.9, 31.7, and 32.2% of worldwide cases respectively, resulting in significantly higher incidence than in high-income regions (Table 1) (Guijon et al., 1992; Lewis, 2011; Rodriguez-Cerdeira et al., 2012; Swanepoel et al., 2013). This issue is exacerbated by the large HIV+, immunocompromised population, which is more readily infected. Screenings of sub-Saharan African women estimate 70–80% of those infected with discharge-causing infections remain asymptomatic (Sylverken et al., 2016; Barnabas et al., 2018). This contributes to increased transmission, but also allows infections to persist for longer before treatment, meaning persistence of a cervical microenvironment conducive to cervical lesions and HPV persistence. Less screened infectious microbes such as *Mycoplasma* spp., *Ureaplasma* spp., and *Leptotrichia amnionii* have been shown to be involved in cervicitis in HIV+ women (Linhares et al., 2000; Dehon et al., 2016; Mitra et al., 2016). Mycoplasma infection has also been directly associated with pre-cancerous cervical lesions (Klein et al., 2019). When screening for STIs, *Mycoplasma genitalium* is more often included, however *Mycoplasma hominis*, which is similar to *M. genitalium* in pathogenesis, is rarely screened for. Because of this, a good estimate of Mycoplasma prevalence in sub-Saharan Africa is not well-established. In sub-Saharan African cohort studies which include *M. hominis* detection, prevalence of *M. hominis* ranges from 17 to 67.5%, far exceeding *M. genitalium* (Agbakoba et al., 2007; Redelinghuys et al., 2013; Kouegnigan Rerambiah et al., 2015; Sylverken et al., 2016). This is significantly higher than what has been shown in North America and may be due to the HIV+ population acting as a reservoir for *M. hominis* infection (Djigma et al., 2011).

### Commensal Microbiota

The cervicovaginal microbiome has been shown to most often fall into one of seven general community state types (CSTs) (Salas and Chang, 2014; Mitra et al., 2016). These CSTs are characterized by the relative abundance of various species of Lactobacillus and anaerobic bacteria and are separated into healthy and dysbiotic groups. The healthy CSTs are all Lactobacillus dominated; type 1 is dominated by *L. crispatus*, type 2 by *L. gasseri*, type 3 by *L. iners*, and type 5 by *L. jensenii*. Common dysbiotic CSTs are characterized by an abundance of anaerobic bacteria; type 6 is dominated by *Gardnerella vaginalis*, type 4 is characterized by a high abundance of anaerobic bacteria and low abundance of *Lactobacillus* species, and type 7 is characterized by high abundance of both *Gardnerella vaginalis* and *Lactobacillus* species. These cervicovaginal CSTs have been previously associated with significantly different prevalences of infecting HPV genotypes (Brotman et al., 2014; Mitra et al., 2015; Dareng et al., 2016; Reimers et al., 2016). *L. crispatus* dominated microbiomes (type 1) are considered to be the most protective against HPV and HIV, and have been shown to be significantly less likely to have HIV, HSV-2, any HPV, or high-risk HPV than other CSTs (Borgdorff et al., 2014). *L. crispatus* produces lactic acid, antimicrobial compounds, and inhibits inflammation (Graver and Wade, 2011; Hickey et al., 2012; Rose et al., 2012; Aldunate et al., 2013; Petrova et al., 2013). These represent likely mechanisms by which *L. crispatus*, and potentially other microbes, are able to influence HPV infection and progression of HPV-associated diseases. A study of Rawandan women found that women with the *L. crispatus* dominated CST had the lowest prevalence of HIV/STIs, with a slight increase in the *L. iners* dominated CST, and a significant increase in the dysbiotic CSTs (types 4, 6, and 7) (Borgdorff et al., 2014). Similar results have been found in studies of Nigerian and South African women, supporting an association between HPV pathogenesis and decreased abundance of *Lactobacillus* spp. (Dareng et al., 2016; Onywera et al., 2019). *L. crispatus* is much less abundant in sub-Saharan Africa than in high-income regions (Jespers et al., 2015). Instead, *L. iners* is the most abundant “protective” cervicovaginal microbe, especially in HIV+ women without dysbiosis. *L. iners* dominated microbiomes have been shown to be less protective against cervicovaginal infections, and closer to dysbiotic CST rates of HPV infection and cervical dysplasia (Norenhag et al., 2020). This suggests that differences in commensal microbiota in sub-Saharan Africa are also contributing to the prevalence and transmission of cervicovaginal infection and dysbiosis in the region.

## Conclusion

**Figure 2:**
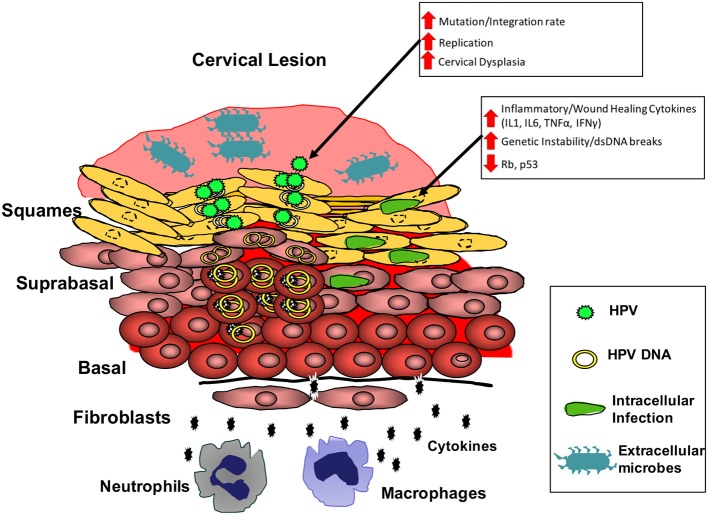
Model for the cervical microbial and immune microenvironment driving cervical cancer. Microbial dysbiosis and infection at the cervical epithelium results in increased local expression of inflammatory and wound healing cytokines (IL1 and IL6). Chronic expression of these cytokines can result in increased genetic instability and reduced tumor-suppressor protein function in infected cells. These conditions increase HPV replication, while also increasing risk of mutation and integration of the HPV genome. Thus, the cervical microbiota can increase the risk for events necessary in the transformation of cells by HPV.

Cervical cancer, a preventable and treatable cancer, remains the cancer with the highest incidence in women in 27 countries, and the leading cause of cancer death in women in 45 countries, most of which are in sub-Saharan Africa. Efforts to determine the most cost-effective strategies to reduce cervical cancer burden through human papillomavirus vaccination and screening are ongoing and will hopefully lead to a continued decrease in cervical cancer incidence in the most affected areas of the world. However, it is expected that the number of women with cervical cancer in sub-Saharan Africa will increase as more women get access to HIV therapy, increasing the life expectancy of HIV+ women (Williamson, 2015). There is therefore an urgent need to roll out better cervical screening programs. To accomplish this, a better understanding of the region's unique factors contributing to cervical cancer is necessary to improve identification and treatment of at-risk individuals before the onset of disease. Current screening and vaccination in Sub-Saharan Africa is sparse, irregular, and does not consider factors beyond HIV and cervical lesion status. In considering the factors contributing to Sub-Saharan Africa's disproportionate burden of cervical cancer, the disparity between HPV prevalence, HPV genotype prevalence, and sociocultural factors in Sub-Saharan Africa and high-income regions is much less than the disparity seen in vaccination and cervicovaginal infection and dysbiosis, suggesting that disruption of the cervicovaginal microbiome may be the most significant factor and predictor of cervical cancer in Sub-Saharan Africa. Research up to this point suggests that latent, basal layer infections of HPV's are activated when sensing the host is immunocompromised, leading to increased HPV replication and development of abnormal cytology. HIV and dysbiosis of the cervicovaginal microbiome are the most common factors in sub-Saharan Africa able to activate HPV via effects on the cervical immune microenvironment. ART may help reduce these effects by restoring immune competence, however it does not return activated HPVs to full latency. Transition to cervical cancer is associated with a single HPV genotype becoming dominant, however data suggests coinfection with multiple HPV genotypes or other cervical pathogens plays an important role early on, potentially contributing to the early pathogenesis of a primary high-risk HPV either directly or through manipulation of the immune microenvironment, leading to increased replication and eventual faster or more frequent development of cervical cancer. This process is likely mediated by expression of inflammatory and wound healing cytokines such as IL1, IL6, TNFα, and IFNγ, leading to cell stress and genetic instability, increasing the risk of mutation or integration of the episomal HPV genome (Figure 2). These infections are likely cleared by a competent immune response, which may explain why multiple infection decreases in HIV- cervical cancer cases, but not in HIV+, immunocompromised individuals. A better understanding of the early events influencing HPV control and persistence in the genital tract is needed to test this theory. In Sub-Saharan Africa particularly, further research on the impact of HIV on these early events is desirable. Identification of the microbial risk factors for development of cervical cancer will allow for improved identification of those at elevated risk, while improving design and application of primary and secondary preventative treatment and screening.

## Author Contributions

All authors helped review literature and write the manuscript. PA revised the figures and manuscript.

### Conflict of Interest

The authors declare that the research was conducted in the absence of any commercial or financial relationships that could be construed as a potential conflict of interest.

## Abbreviations

NILM: Normal for intraepithelial malignancy
HPV: human papillomavirus
HIV: human immunodeficiency virus
ART: antiretroviral therapy
CST: community state types
STI: sexually transmitted infection
VIA: visual inspection with acetic acid
PID: pelvic inflammatory disease
BV: bacterial vaginosis.
